# Masking the Integration of Complementary Shape Cues

**DOI:** 10.3389/fnins.2019.00178

**Published:** 2019-03-13

**Authors:** Andrew Geoly, Ernest Greene

**Affiliations:** Laboratory for Neurometric Research, Department of Psychology, University of Southern California, Los Angeles, CA, United States

**Keywords:** visual persistence, visual masking, working memory, shape perception, shape integration

## Abstract

Retinal and cortical mechanisms provide for persistence of visual information across intervals of many hundreds of milliseconds, which supports the integration of partial shape cues. The present experiments displayed unknown shapes in a match recognition task, wherein a target shape was quickly followed by a comparison shape; the task was to specify whether the comparison shape was the same or different from the target. The target and comparison shapes were displayed as sparse dots that marked boundary locations. The first experiment successively displayed the target shape as two complementary subsets and found that the probability of correct match remained above chance with up to 500 ms of subset separation. The second experiment demonstrated masking of the target by a random pattern of dots when the target and mask were displayed simultaneously, but with much less or no masking when the two were separated by 100 ms. The third experiment displayed the target subsets with 200 ms of separation and found that match recognition was disrupted when the random-dot mask was displayed midway between the two subsets. Much less masking of an intact target was produced with that amount of temporal separation, which suggests that mechanisms for integration of shape cues have a special vulnerability to masking. The third experiment also found very little impairment of match recognition when the mask was displayed simultaneous with one of the subsets. We hypothesize that there is embedding of the subset pattern within the mask pattern, but additional display of the other subset effectively disembeds the buried partial shape cues.

## Introduction

“[T]here is some support for the view that sensory persistence is produced by the activity of coding mechanisms at the level of feature extraction in visual information processing”.

*Vincent* Di Lollo (1977).

A substantial amount of work has been done to evaluate early stages of shape encoding, with masking and manipulation of neuronal persistence providing some of the most effective research tools. It seems odd, therefore, that we have very little information about the effect of masking on the persistence of stimulus information. Persistence across several 100 ms is thought to mediate working memory, and masking has been used to manipulate the contents of working memory, so it is a reasonable hope that the combined use of both methods would provide useful insights about the nature of working memory.

**FIGURE 1:**
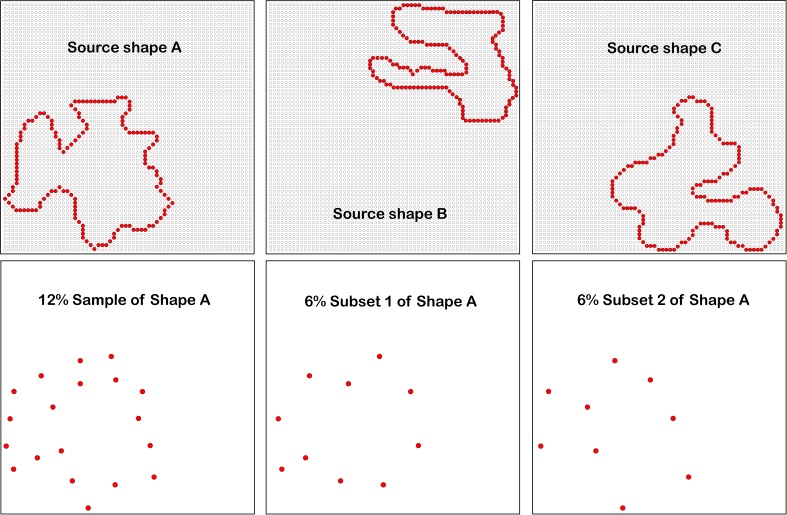
Three examples of source shapes are shown in the upper panels, with the dots of the display board provided as background. The experiments displayed low density patterns that were sampled from source shapes, providing either 12% sets or successive displays of complementary 6% subsets. For ease of discourse, the term “shape” will also be used to describe these patterns. For better visibility, the background dots of the array are not shown in the three lower panels, and dot size has been increased.

The research reported below differed from prior research in fundamental ways. First, the targets to be identified were unknown shapes, each seen only once using a match-recognition protocol. A given source shape consisted of a sequence of dots that marked an outer boundary, similar to an outlined figure (see Figure 1). The experiments displayed reduced-density versions of these shapes, meaning that a sparse subset of boundary markers provided the target to be identified. Following display of a given target, a sparsely marked comparison shape was displayed that either matched the target or provided a non-matching shape. Because each target shape was shown only once to a given respondent, all identification was based on short-term memory.

Second, the display equipment avoids some of the problems that have bedeviled the study of temporal integration of visual cues, which can also be described as information persistence (Coltheart, 1980). Much of the earlier work has used cathode ray tubes with short-lived phosphors to display the stimuli, so that any persistence could be correctly attributed to the visual system rather than the display itself. A fast phosphor can fall to about 1% residual emission within microseconds, but it can then persist at that level for a second or more. Rayner and Pollatsek (1983) demonstrated that observers could still perceive this weak afterglow, providing a basis for combining successive displays using persistence of the stimulus itself. Jonides et al. (1983) failed to replicate earlier work done in their own lab when the displays were presented with LEDs rather than with phosphor-based images (see also Di Lollo et al., 1997, 2000). The present work avoids these issues by using an LED array to display the shape stimuli. Also, the equipment can display successive dot patterns with microsecond control of timing for pulse duration and interstimulus interval.

Initial experiments provided confirmation that classic information persistence and masking effects would be found with this match-recognition task. Then the influence of masking on temporal integration was evaluated.

The target shape was divided into complementary subsets that were successively displayed, which requires the integration of shape information across an interstimulus interval spanning hundreds of milliseconds. Then the ability of a mask to impair the integration process was examined. The results suggest the possibility of a motion-to-form encoding mechanism as part of working memory, as will be discussed subsequently.

Visibility of a briefly displayed image can be reduced by presentation of a different image, which we describe as visual masking. Generally, one image provides the target, with the task requiring discrimination or recognition, and the mask acts to impair effective performance of the task. The relative timing of target and mask can determine whether visibility is affected. The experimental protocol is designated as *forward masking* if the mask precedes the target, *backward masking* if it follows the target, and *concurrent (simultaneous) masking* if the two are presented at the same moment (Breitmeyer, 2007).

The earliest masking studies used large, spatially uniform increments of luminance (Baxt, 1871; Crawford, 1947; Sperling, 1965). Subsequent work often has used masks that contained image elements, such as shapes, lines, or dots (Rieger et al., 2005). The term *pattern masking* serves as a general classification of masks that provide these components (Enns and Di Lollo, 2000), irrespective of whether there is temporal separation, i.e., backward, forward, or simultaneous display. Impaired discrimination or recognition of the target has been attributed to inhibitory interactions among neurons that register the image elements (Weisstein et al., 1975; Breitmeyer and Ganz, 1976; Macknik and Livingstone, 1998).

There are subcategories relating to the spatial attributes of pattern masks. For instance, noise masks could be made up of dots or boxes that have little in common with the target image (Kinsbourne and Warrington, 1962). Alternatively, structure masks would be those that bear a strong resemblance to the target, or have lines with common orientations. For example, a pattern composed of lines might be used to mask alphabetical letters. Specific pattern masks can vary with respect to contrast, luminance, or other physical parameters, limited only by the creativity and discretion of the investigator.

Three different mechanisms for masking are often invoked, one being the *erasure* of stimulus information, another calling for *integration* (merging) of stimulus information, and the third providing for *interruption* of perceptual processing. Each may be acting within the retina or in cortex. Persistence of retinal activity in *integration* masking can cause the target and mask to be perceived as a unitary pattern that precludes recognition or discrimination of the target itself (Eriksen, 1966; Turvey, 1973; Breitmeyer, 1984). This model seems most plausible when one gets maximal disruption of performance with simultaneous display of target and mask, and one perceives the combined image of the target embedded in the mask pattern. It is common to see the masking effect become nil with about 100 ms of target/mask separation in either direction (Enns and Di Lollo, 2000).

Alternatively, pattern masking can occur with substantial temporal separation of target and mask through *interruption* of information processing (Bachmann and Allik, 1976). Because the mask can act across an extended interval, most attribute the masking to disruption of cortical mechanisms that are required for recognition or discrimination. Alternatively, one might see a U-shaped function wherein there is progressive impairment of performance across an extended range of target/mask separation, followed by recovery of target recognition or discrimination (Bachmann and Allik, 1976; Michaels and Turvey, 1979).

Conditions that produce a delay of target masking have been designated as *metacontrast masking*. Here the mask consists of an annulus that surrounds the target image, and masking only occurs within a narrow temporal range (Enns and Di Lollo, 2001). When the target and mask are simultaneously displayed, or the interval between them is very short, the target is clearly visible and is seen as lying within the interior of the mask. With very long separations the mask is ineffective at impairing shape processing, and both are visible. At intermediate intervals, perception of the target is impaired, yielding a U-shaped function of accuracy (hit rate) as a function of the separation interval (Enns and Di Lollo, 2001). One explanation for the effect, which can be described as a “two-channel” theory, proposes that image information is transmitted by a fast burst of neuronal activity followed by sustained (tonic) activity that conveys fine details about the stimulus attributes. A metacontrast mask is thought to occur when the fast-acting signals from the mask’s onset interfere with the sustained slower signals of the tonic channel, disrupting the processing information about the earlier target.

The first experiment was a replication of a Greene and Hautus (2018) experiment that examined temporal integration of shape cues for unknown shapes in a match-recognition protocol. The shape cues were provided by sparse dots that marked the outer boundary of a given shape. This is a new experimental approach to the study of shape recognition, so it is appropriate to show that the integration of shape cues over a span of half a second is a reliable finding. This is especially worthwhile given current concerns about reproducibility of results.

The second experiment examined mask interference with the integration of unknown shapes, wherein the mask consisted of a random pattern of dots. The masking stimuli were random dot patterns, which seems especially appropriate for either overwriting the information from sparse markers, or interrupting short-term memory of that information. The experimental results provided evidence for classic disruption of shape recognition when the target and mask were simultaneously displayed, but the mask was relatively ineffective when it was separated from the shape cues by about 100 ms.

The third experiment examined mask interference with temporal integration of the shape cues. Based on findings from Experiment 2, there was an expectation that simultaneous display of the mask with a subset of the shape cues would greatly impair match recognition. Further, there was an expectation that there would be no interference with match recognition where the temporal separation of mask from the shape cues was 100 ms, as was found in Experiment 2. Neither expectation was confirmed, i.e., the results were the opposite of expectation. These findings suggest some new principles for how stimulus information is integrated and stored in working memory, which will be discussed once the experiments have been reported.

## Materials and Methods

### Authorization and Consent of Respondents

This study was carried out in accordance with the recommendations and guidelines of the Psychology Department Subject Pool. The protocol was approved by the USC Institutional Review Board. All respondents gave written informed consent in accordance with the Declaration of Helsinki. A total of 24 undergraduates volunteered and provided data, eight for each of the three experiments reported below.

### Source Shapes, Sets, and Subsets

An inventory of 480 unknown shapes provided the source of stimulus patterns that were displayed in each of the three experiments, so hereafter they will be described as “source shapes.” Each source shape consisted of a continuous string of dot locations on a display board (detailed below), forming a shape boundary akin to a silhouette. The number of dots in source shapes ranged from 100 to 269, with the mean being 168 dots. Distance from the centroid to dots ranged from 12.8 to 22.3 dots, mean distance being 16.9 dots. The shapes were constructed to avoid similarity with known shapes and objects, to avoid long-term memory factors and to focus on early sensory encoding. Examples of three of the source shapes are shown in the upper panels of Figure 1.

The three experiments displayed low-density samples drawn from source shapes, which can be designated as “sets” and “subsets.” We can characterize these dot patterns as “shapes,” with the understanding that they are providing various degrees of effective cues relative to the original shapes from which they were derived.

Shape sets were at 12% density and the subsets provided complementary 6% densities. The dots for a given 12% set were chosen by first randomly picking a starting point from among the boundary dots of the source shape, and then proceeding along the boundary, marking every eighth dot to be included for display. The final dot that was chosen at the end of the circuit would commonly leave a span that was shorter than the others. The computer made these selections “on the fly,” meaning that the dots displayed to a given respondent were chosen at random on each trial.

For the experiments that displayed complementary 6% subsets, the 12% set was further divided. Beginning at a randomly chosen starting point, dots were successively numbered. The odd numbered dots were designated as “subset 1” and the even numbered dots were assigned to “subset 2.” The fact that the two subsets can be combined to provide a 12% set is the basis for describing them as being “complementary.”

### Mask Stimuli

Experiments 2 and 3 included display of random-dot masks to evaluate the conditions that would impair match recognition of the targets. A 4% dot-density level was chosen to provide approximately the same number of dots in the mask as the mean number of dots among the shapes in the inventory. To be specific, the mean number of dots in the inventory of shapes is 166 (100 at the minimum and 269 maximum), and a 4% random sample from the full LED array provides 164 dots. For trials in which a set or subset with displayed simultaneous with the mask, the random selection of dot locations did not include the locations of set or subset dots. A different random pattern was used on each trial in which a mask was presented.

**FIGURE 2:**
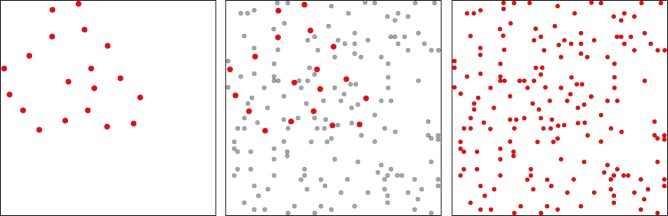
The left panel illustrates a low-density shape set, i.e., a 12% sample of dots from Shape C in Figure 1. The middle panel shows a 4% random-dot pattern that could mask of the shape set. Here the mask dots are shown in gray so that one can still see the dots of the 12% set (in red). Dot sizes in the set have been enlarged in both panels to provide for better discrimination of the shape set in relation to mask dots. The right panel has colored all dots red, with mask and shape-set dots rendered as the same size. This illustrates the stimulus that would be seen with simultaneous display of the mask and the 12% shape set. One can see that the mask precludes perception of shape boundary markers, and the same would be true for the 6% subsets, which provide only half the number of markers.

Figure 2 illustrates how the random-dot pattern is effective at precluding perception of low-density shape samples. The left panel shows a 12% set derived from Shape C of Figure 1. The middle panel shows a 4% mask superimposed on the set. Here the mask dots are shown in gray so that one can still pick out the locations of dots in the 12% set. The right panel shows all the dots in red, this being the stimulus that the respondent would see with simultaneous display of the mask and shape set. It is clear from inspection that the 12% set cannot be discriminated in the presence of the random-dot pattern, which assures that the mask would be effective in impairing match recognition. The 4% random-dot pattern would also mask information from display of complementary 6% subsets, which together would be equivalent to the 12% set.

### Stimulus Displays

Room illumination was dim (10 lux). Shape and mask patterns were displayed as brief flashes on a 64 × 64 array of LEDs mounted on a display board. All dots of a given pattern were displayed as simultaneous ultra-brief flashes. Each flash had a duration of 10 μs and an intensity of 1000 μW/sr. At a viewing distance of 3.5 m, the visual angle of a given dot of the display board was 4.92 arc°, dot to dot spacing was 9.23 arc°, and the total span of the 64 × 64 array (horizontal and vertical) was 9.80 arc°. The shape patterns that were displayed would therefore have overall dimensions that ranged from 2.0 to 3.5 arc°, with the mean being 2.6 arc°.

### Basic Task Conditions

The basic task can be described as requiring a match-to-sample judgment, which for convenience can be described as match recognition. When used with unknown shapes, one can assess the encoding and persistence of shape information without the confounding influence of long-term memory (Greene and Hautus, 2017, 2018).

The present work calls for initial display of a “target” set (or subsets), followed by display of a “comparison” set that might or might not be the same shape as the target. On each trial, target and comparison shapes were chosen at random from the inventory. A given shape was shown only once as a target, or only once as a non-matching comparison shape. On half the trials the comparison shape was the same as the target shape, which was designated as “matching,” and on half it was a different shape, designated as “non-matching.” In other words, the task is asking whether the cues provided by the target set or subset are sufficient for recognition of the comparison set.

The order of treatments was chosen at random. The corner in which the target was displayed was chosen at random on each trial in each of the experiments. The comparison set was then displayed in one of the other three corners, again chosen at random. Positioning of a given pattern required placement of at least one dot in the outside boundary of the top or bottom, and one dot on a side edge of the array.

A fixation point consisting of four central dots was provided prior to each trial, and respondents were instructed to keep their eyes centered on this location. Following each display sequence, the respondent voiced a decision of whether the comparison shape was the “same” or “different” from the target shape, and this information was entered by the experimenter into a computer file. Neither the experimenter nor the respondent was informed as to which treatment condition was presented on a given trial, or whether the judgment was correct.

### Experimental Treatments

**FIGURE 3:**
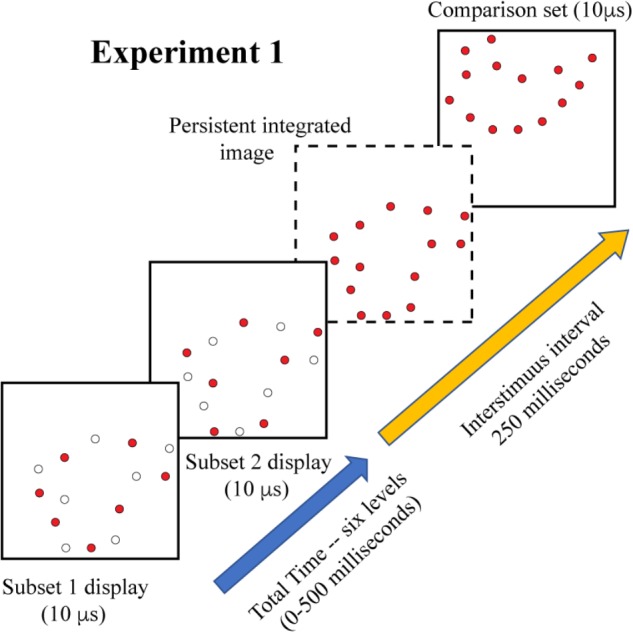
On each trial of Experiment 1, a randomly sampled shape provided the complementary subsets as a target. The frames show the displayed subset as colored dots, and include open dots to show which members of the 12% pattern would not be displayed, i.e., they remain dark at that moment. Display of subset 1 was followed by subset 2 at an inter-stimulus interval that varied from 0 to 500 ms. If the shape information from the two displays were completely integrated by the visual system, the resulting image would contain 12% of the boundary markers, as illustrated in the frame shown with broken lines. This amount of shape information would be expected to provide a moderately high level of shape identification. A comparison shape was shown 250 ms after display of the second subset, providing an opportunity for a shape-matching decision.

Experiment 1 displayed complementary 6% subsets as targets, with six levels of inter-stimulus interval, specifically: 0, 100, 200, 300, 400, and 500 ms. The comparison shape. either matching or non-matching, was displayed after an additional interval of 250 ms. The display sequence is illustrated in Figure 3. Each subject judged 25 trials for each of these treatment conditions for a total of 300 trials.

**FIGURE 4:**
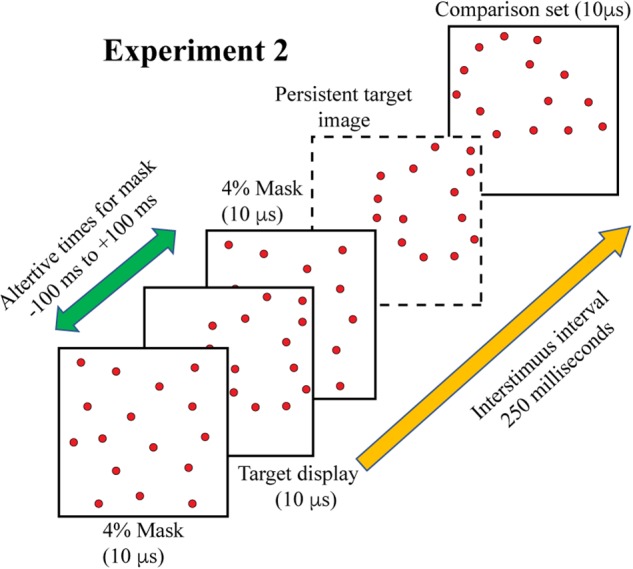
On each trial a full 12% target shape was flashed, followed 250 ms later by the comparison shape. In addition, a 4% random-dot mask was displayed at times that either preceded, followed, or was simultaneous with the target display. The illustration shows the 4% mask twice to represent the two ends of the range at which it could be displayed. The dashed frame shows a target shape that was unaffected by the mask, though this would not be expected if the mask erased the stimulus information, occluded it, or otherwise interfered with shape processing.

Experiment 2 examined masking of target-shape information. All targets were displayed with 12% density, this being to demonstrate effectiveness of masking against integrated 6% subsets (in Experiment 3, to follow). A 4% random-dot mask was added to each target-comparison sequence, with display of the mask coming either before, during, or after display of the target set. The mask/target intervals were: −100, −50, 0, +50, +100 ms – the negative values designating display of the mask prior to the target and positive values designating display of the mask after the target. At 0 ms the mask dots were displayed at the same 10 μs moment, so the dots of both patterns were superimposed, as illustrated in Figure 2. The inter-stimulus interval between target shapes and comparison shapes was again 250 ms. Figure 4 illustrates the display timing. Each subject judged 32 trials for each of the five treatment conditions for a total of 320 trials.

**FIGURE 5:**
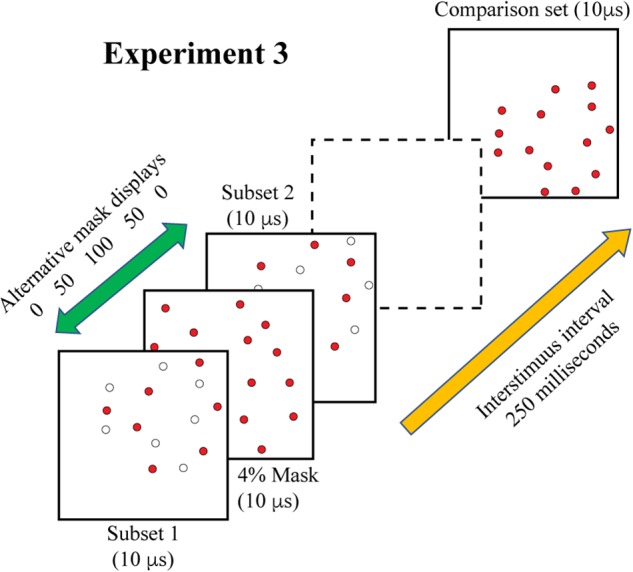
In Experiment 3 the two complementary subsets were displayed 200 ms apart, with the comparison shape being provided 250 ms after the second subset was shown. A 4% random-dot mask was also provided, either simultaneous with subset 1, simultaneous with subset 2, or at three intervals in between the subset displays. We are designating the midpoint of this range as 100, for this would display the mask 100 ms away from each subset. The goal was to determine whether information from the two subsets would be integrated or if the mask would impair this process. This illustration shows the mask precluding effective integration, i.e., impaired match recognition, in that the dashed frame does not contain a persistent image of the combined subsets. Two additional control conditions were included in the experiment (see text).

Experiment 3 combined treatments that would require integration of target cues as well as masking of those cues. On each trial, the two 6% subsets were displayed with a temporal separation of 200 ms. A 4% mask was inserted into this sequence to provide the potential for disruption of the integration process. For logging of data and statistical analysis, mask timing was specified relative to the first subset display, i.e., at 0, 50, 100, 150, and 200 ms. However, the mask was expected to produce disruption of performance when simultaneously displayed with either of the subsets, and provide the least influence of judgments at the midpoint between display of the subsets. Therefore, we have re-designated the treatment levels as 0, 50, 100, 50, and 0, providing labels that reflect two symmetrical limbs of mask influence. These display conditions are illustrated in Figure 5.

Two additional control treatments that did not include a mask stimulus were added to provide measures that aid in interpreting effects. One control treatment displayed just the two subsets, which provided evidence of performance from simple integration of the two sources of shape information. The other control treatment displayed only one of the subsets, this to establish the level of performance if there was masking of information from only one of the subsets. As in each of the earlier experiments, the comparison shape was presented 250 ms after display of second subset. Each of the five mask and two control conditions were displayed to a given subject for 22 trials, for a total of 308 trials.

### Bias Correction of Judgments

Responses were evaluated with signal detection analysis that derived an unbiased index of performance. Signal detection theory provides a framework that corrects for response bias. A method developed by Macmillan and Creelman (2005) was adopted, which uses the bias-correcting formula *d*′ = $\sqrt{2}$(*z*(*H*) − *z*(*F*)). In this formula, H is the proportion of “same” judgments to matching shapes, F is the proportion of “same” judgments to non-matching shapes, and *z*(•) is the inverse-normal transform (Green and Swets, 1966/1974) Values of F and H were adjusted for values of 0 or 1 (which would otherwise lead to *d*′ = ±∞) prior to calculation of d’. We adopted the log-linear correction for this purpose (DeCarlo, 1998). Bias correction requires the combination of response information from both matching and non-matching shapes.

It is more intuitive to express performance as a proportion, which can be done by converting d’ into *p(c)*_max_ using the formula:

$$p{\left(c\right)}_{\max}=\Phi \left(d^{\prime }/\left(2\sqrt{2}\right)\right)$$

Here the function _Φ_(•)__ is the cumulative distribution of the normal distribution. The *p(c)*_max_ index scales with 0.5 being chance and 1.0 being decisions that are perfectly correct. For convenience, the present discourse will describe this index as “probability of match recognition.”

### Statistical Analysis

For each of the experiments, linear mixed-model regression was used to test for omnibus treatment effects. Experiment 1 was a replication of earlier work and there exists a substantial body of literature on masking effects, so we had clear expectations about the influence of treatments for each of the three experiments. This justified the use of planned comparisons to test hypotheses about specific treatment effects. For Experiments 1 the only test of interest was whether the mean at the longest temporal separation would be above chance. For Experiment 2 the question was whether performance would be above chance where the mask and shape subset were displayed simultaneously.

Experiment 3 provided results that were the opposite of what was predicted, so *post hoc* tests of mean differences were done instead of planned comparisons. Specifically, we tested whether each mean that was observed with simultaneous display of mask and shape subset differed from the control condition that displayed the subsets with no mask being present. We also tested whether masks presented midway between the two shape subsets, i.e., at 100, differed from the one-subset control condition, and whether it differed from chance.

A piece-wise linear regression was calculated to assess the influence of temporal separation of the mask from each subset. Based on the results of Experiment 2, there was an expectation that the mask would block shape information when it was display at the same moment as the subset, and would have little or no effect when it was separated from the subset by 100 ms. Therefore, the plan was to do a separate regression analysis for each leg of the sequence, i.e., from 0 (mask + subset 1) to 100, and then from 100 to 0 (mask + subset 2) – see Figure 5, 8. The regression itself makes no prediction about the direction of effect, so this was still the appropriate analysis even though the results were opposite of what had been expected.

## Results

### Experiment 1

**FIGURE 6:**
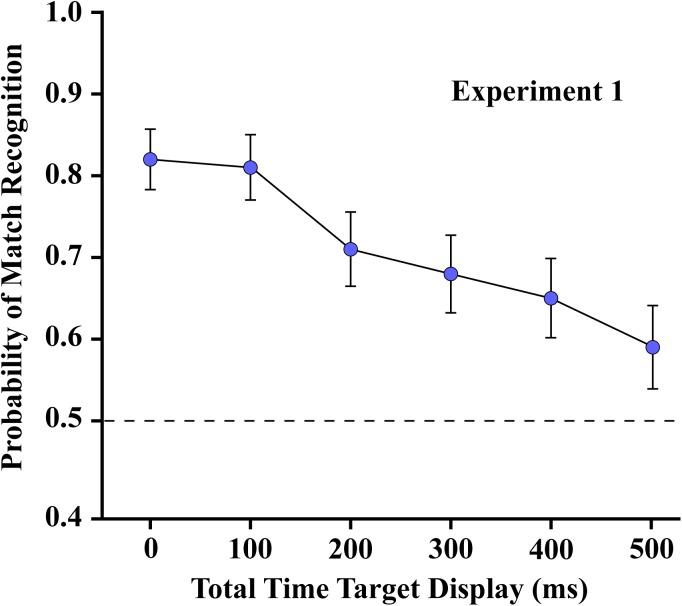
The mean probability of match recognition for the respondents of Experiment 1 are plotted for each of the six levels of temporal separation of the two subsets. Judgments were well above the chance level of 0.5 with up to 500 ms of separation.

As shown in Figure 6, the probability of correct matching decisions was quite high (above 0.8) where the two subsets were displayed simultaneously, i.e., with temporal separation of zero. Performance dropped as the interval between the two subsets was increased, which reflects a decline of persistence of shape information from the first subset. Regression across the five treatment levels confirmed that the decline was significant at *p* < 0.0001 (slope = −0.0005/ms, t_7_ = −9.37).

The probability of match recognition was still well above chance at the longest subset separation interval (500 ms). A planned comparison established that the mean at the 500 ms treatment level was significantly different from chance (*t*_35_ = 2.92, one sided *p* = 0.0031). That level of performance was about the same that found in Experiment 3, where a control condition provided display of a single subset (see below). It is likely, therefore, that by 500 ms all shape information from the first subset had completely evaporated, providing a level of match recognition that could be elicited by the second subset, acting alone.

### Experiment 2

**FIGURE 7:**
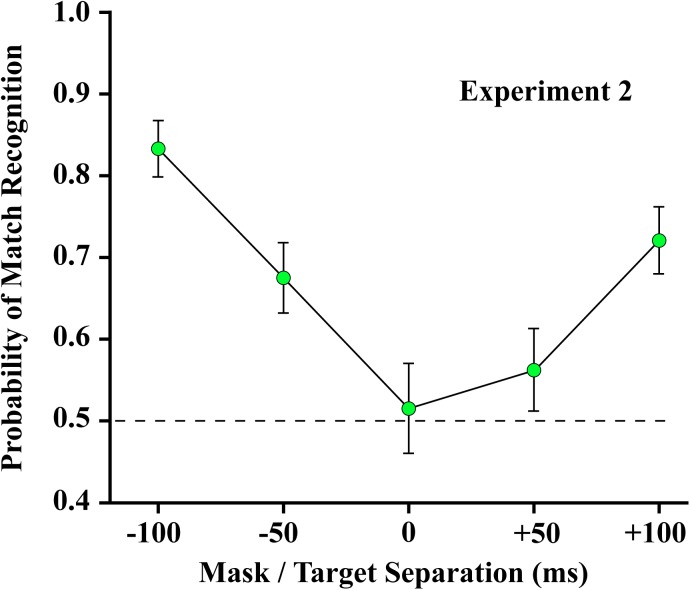
The 4% mask was ineffective at blocking match recognition when it preceded the 12% target by 100 ms and was only partially effective when it led by 50 ms. Match recognition was at chance levels when the mask and target were displayed at the same instant (designated as 0 ms). Judgments were above chance when the mask followed the target by 50 ms or more.

The results of Experiment 2 are shown in Figure 7. Linear regression confirmed a significant decline in forward masking between −100 and 0 ms (slope = −0.0032/ms, *t*_23_ = −8.74, *p* < 0.0001). Match recognition was well above chance when the mask preceded the target by 50 ms, and no masking was evident with a temporal separation of 100 ms. Trends were similar when the mask followed the shape set (slope = 0.0020/ms, *t*_23_ = 5.46, *p* < 0.0001), though masking was less complete at the longest interval. These results suggest that greater proximity of mask and target is needed for forward masking to be effective than is required for backward masking. The mean at 0 ms of mask/target separation was not significantly different from chance (*t*_28_ = 0.49, unadjusted *p* = 0.6273). [Where the mean does not differ significantly from chance, an unadjusted comparison is the more conservative statistic].

### Experiment 3

**FIGURE 8:**
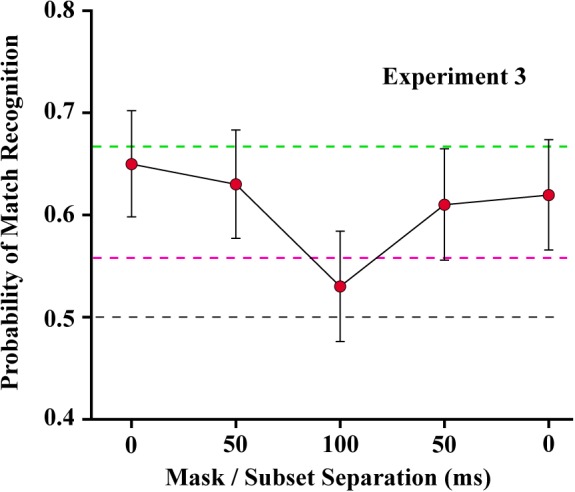
Mean probability of match recognition judgments are plotted for the masking conditions of Experiment 3. The mask was ineffective at blocking match recognition when it was displayed simultaneous with one of the subsets, i.e., at either 0 position. At ±50 ms of separation from a subset the mask was only partially effective. At 100 of separation, midway between the two target subsets, match recognition was not greater than chance. The green dashed line indicates the level of match recognition from successive display of the two subsets, wherein there was no mask in the display sequence. The red dashed line indicates the level of match recognition when only a single (6%) subset was displayed.

For Experiment 3, piece-wise linear regression found a significant decline in match recognition as the mask was temporally separated from each of the shape subsets (see Figure 8). The decline with mask separation from subset 1 (from 0 to 100) was significant at *p* = 0.0031 (slope = −0.0011/ms, *t*_30_ = −3.22), and with separation from subset 2 (from 100 to 0) was significant at *p* = 0.0205 (slope = 0.0008/ms, *t*_30_ = 2.45). Simultaneous display of mask and shape subsets yielded means that were not significantly different from the first control condition (green broken line), wherein the two subsets were displayed without any mask being provided (*t*_42_ = −0.52, unadjusted *p* = 0.6054 and *t*_42_ = −1.13, unadjusted *p* = 0.2632 for subset 1 and subset 2, respectively). The mask at treatment level 100 – midway between the shape subsets – did not differ significantly from the second control condition (red broken line) that assessed match recognition with display of a single subset (*t*_42_ = −1.11, unadjusted *p* = 0.2750).

## Discussion

Visible persistence is the continued perception of a sensory image, like an afterimage (Coltheart, 1980), and is thought to reflect persistence of neuron activity within the retina. It is quite sensitive to the physical parameters of a stimulus, given that increasing the luminance of a stimulus will cause the period of visible persistence to decline (Dick, 1974). Also, it is quite vulnerable to simultaneous masking or backward masking protocols that have short inter-stimulus intervals (Long, 1980). A bright, spatially uniform flash can readily disrupt processing of image content (Baxt, 1871; Crawford, 1947; Sperling, 1965). It is generally thought that masking of visible persistence occurs within a 100 ms window (Coltheart, 1980; Greene and Visani, 2015), so the simultaneous display of the mask with the 12% target (Experiment 2) would have intruded upon visible persistence of the boundary markers.

In Experiment 2 the 4% random-dot mask likely embedded the 12% targets when the two were presented at the same moment, so the shape could no longer be identified as the same or different from the comparison shape that was subsequently displayed. The random-dot mask was ineffective at blocking match-recognition when it preceded the target shape by 100 ms, and performed slightly better when it followed the target shape. These results are consistent with previous findings (Schultz and Eriksen, 1977).

If the stimulus is sufficiently salient, it can go out from the retina very quickly (VanRullen and Thorpe, 2001). If the separation of pattern and mask is 100 ms or longer, one can be fairly sure the image information has been passed to cortex and any masking disrupts retrieval of information from working memory or long term memory (Kolers, 1962; Turvey, 1973; Enns and Di Lollo, 2000; Vogel et al., 2006).

Information persistence is a longer duration process that mediates encoding of stimulus information (Coltheart, 1980; Greene, 2007). The duration of information persistence grows longer as the duration of stimulus display is increased (Irwin and Yeomans, 1986). Numerous laboratories have reported persistence of visual information for many hundreds of milliseconds (Sperling, 1960; Eriksen and Collins, 1968; Hogben and Di Lollo, 1974; Coltheart, 1980; Chun and Potter, 1995; Ward et al., 1997; Vogel et al., 2006). Some of the evidence was based on what can be called the “temporal integration” paradigm, where stimulus information is divided into complementary subsets and the time required to integrate the information is assessed. Greene and Visani (2015) displayed letters composed of dots that were divided into complementary subsets, as in the present work. There was substantial summation of information from the two displays for 200 ms, after which the hit rate remained above the one-subset level across the treatment range (one second being the longest that was tested). Greene (2016) found that complementary-dot subsets provided for persistence of information for recognition of “thin” letters for upward of 600 ms.

Letters are extremely overlearned and the number of potential alternatives are relatively small, thus it should not be surprising that the choices could remain correct on the basis of minimal information, making it possible to observe an extended duration of persistence. Earlier work had suggested that shape information will decay much faster. Greene (2014) used a temporal integration protocol where the task called for recognition of diverse real-world shapes, e.g., animals, plants, vehicles, tools, furniture. Here the boost provided by the temporal integration condition declined to the one-subset level within 100 ms. For this task the information to be retrieved was extremely open-ended, requiring comparison of shape cues against an indeterminate store of shape memories. Singer and Kreiman (2014) found similar results for integration of image patches where the task called for specifying the category of the objects being shown. Asking to identify the outline of a real-world object may require substantially more information, meaning that even modest decay of the information could preclude effective recognition.

The present work used unknown shapes, each being displayed only once, so decisions were not based on retrieval of information from long-term memory. Match recognition declined as a function of time, following a near-linear trajectory, but remaining well above chance across the 500 ms range that was tested. A prior report from this laboratory found similar results (Greene and Hautus, 2018). We are confident that the temporal integration protocol – the display of complementary subsets – calls for integration of shape information in working memory, and this integration can be provided across intervals of several 100 ms. This finding is critical in interpreting the findings in Experiment 3, which yielded very unexpected results.

Pattern masks, which would include the 4% random-dot mask used here, can work by erasing, embedding, or overwriting the shape information that might otherwise be discriminated or recognized. In Experiment 3 the target pattern was divided into two complementary subsets that were displayed with a 200 ms separation. Displaying the mask in the middle of this interval, with a 100 ms of separation from either subset, yielded performance that was statistically within the chance range. In Experiment 2 the mask was completely ineffective when it followed the 12% target by a 100 ms and was substantially ineffective when it preceded the target by that much. A follow-up experiment using a 6% target found that recognition was at chance levels when the mask preceded the target by 100 ms (unreported data). Therefore, there should have been no impairment of performance in Experiment 3 where the mask was separated from both subsets having 6% density by 100 ms, designated as zero in Figure 8. Yet this condition produced the greatest level of masking, with performance being in the chance range.

Apparently, with 100 ms of separation the mask does not greatly impair fully integrated shape information that is being held in working memory (Experiment 2) but does impair the processing of information that is being integrated, as was the case for the temporal integration required in Experiment 3. We infer that the integration of shape information is a special state that is more vulnerable to masking. As a potentially related matter, Greene and Hautus (2018) found the decline in match recognition across a 500 ms interval was more rapid when two subsets were being integrated than when the dots of the target were displayed one at a time. Apparently having a higher complement of boundary dots available at a given moment can foreclose the integration process, and once completed, the summary is less subject to decay or disruption. This result might be attributed to object substitution masking (Enns and Di Lollo, 1997), wherein the information from the first subset is lost and hence no integration is possible between the first and the second subsets.

**FIGURE 9:**
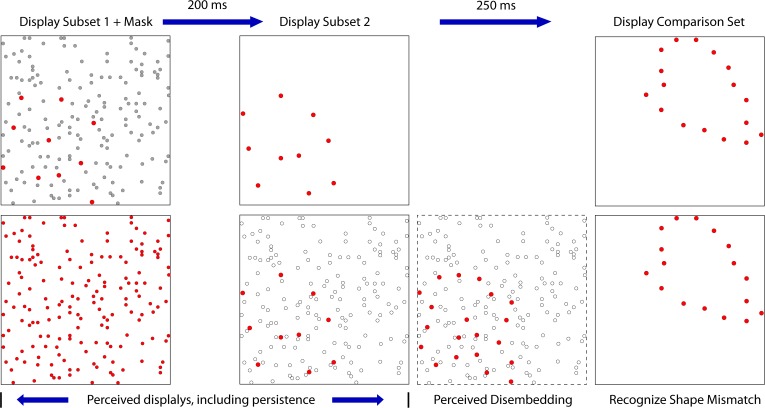
This figure illustrates the disembedding concept where the mask is presented simultaneously with subset 1. The upper panels show the stimulus configurations, providing subset dots as red and the mask dots as gray. The lower panels illustrate the perceptual states that are hypothesized. The first panel on the left shows the first subset as being buried within the mask dots, which prevents the pattern from being perceived. The second panel shows a decreased salience of mask dots due to decay of persistence, so the newly flashed dots of the second subset are conspicuous. The third panel illustrates the second subset disembedding the dots of the first subset, making the full target set available for match recognition. A similar process is assumed for simultaneous presentation of the mask with the second subset.

Experiment 3 also displayed the mask simultaneously with the 6% patterns of subset 1 or subset 2. This should have completely precluded any use of shape information from the masked subset, given that simultaneous masking of a 12% target produced chance performance (Experiment 2). Instead, masking of subset information was relatively weak, and match recognition was well above chance. These results suggest that the subset pattern becomes embedded in the random dot pattern of the mask, and the other subset is able to disembed the subset pattern from the mask. Apparently this can occur in either direction. So where the mask was superimposed on subset 1, the subsequent encounter with subset 2 accomplished disembedding of the target information. And where subset 1 was displayed alone, it persisted across the 200 ms and was able to disembed the subset 2 pattern from the mask. Figure 9 illustrates this hypothesis.

We previously noted that a mask can impair recognition through erasure, integration (embedding), or disruption of process. We now have evidence that a partial target pattern that is embedded in a noise mask can be disembedded by display of the remaining target information. The process of disembedding a subset from a background might be attributed to feature salience of the subset pattern. But perhaps it is a product of form-based motion processing, which would be consistent with much of the current literature relating to dynamic object perception. Finding that simultaneous masking of a subset did not greatly impair match recognition is consistent with conclusions by McCarthy et al. (2017) that simultaneous events should not be integrated if they do not appear along the same contour. Consequently, when the second subset is presented, it may be interpreted as a rotational or translational continuation of the first subset — irrespective of the mask — and vice versa.

It has been postulated that although an object’s identity is maintained during occlusion, its specific features might not be. The continuity seen across objects that disappear behind a source of occlusion and then re-emerge may have more to do with spatio-temporal continuity than spatial continuity (Erlikhman and Caplovitz, 2017). A case can be made that a temporal integration paradigm with an interleaved mask emulates this same type of occlusion and re-emergence, which would explain the results in terms of similar spatio-temporal continuity.

The disembedding concept that is illustrated is Figure 9 might relate to motion-form cueing. A real-life example might be seen where a dog runs behind a white picket fence. The dog’s features are broken up into subset components (in the slits between the boards), none of which would be recognizable as a dog. But on seeing the complements of each subset as a sequence of cues, the viewer perceives a dog running behind the fence. The processes of disembedding may be akin to this kind of motion cueing.

Interestingly, some reports of brain mechanisms are consistent with the current results. An interaction between the dorsal and ventral visual pathways is thought to underlie form motion interactions, in particular the updating of “no-longer-visible” information (McCarthy et al., 2017). Processing form and motion information calls for activity from a number of brain structures, including V3A, V3B, Kinetic Occipital cortex (KO), Medial Temporal cortex (MT), and the inferior parietal sulcus. This system may provide mechanisms for deriving “structure from motion” (Klaver et al., 2008) “biological motion” (Vaina et al., 2001), processing of motion edges (Vinberg and Grill-Spector, 2008) and contour curvature during rotational motion (Caplovitz and Tse, 2007). Moreover, in an experiment to investigate what information is represented during dynamic occlusion, Erlikhman and Caplovitz (2017) measured BOLD fMRI activity across both early (V1–V3) and higher-level cortical areas while observers viewed various shapes passing behind occluding quadrants. They found that that the information represented in early visual cortex during dynamic occlusion is not shape-specific. Rather, it may correspond to the object’s position, its motion path, or the path of attention. Further analysis found that shape identity could be decoded in higher visual areas such as VO, LO, TO, LOC, PHC, parahippocampal place area, and hMT. We hope our research may aid in understanding the interaction between dorsal and ventral stream pathways that have a role in dynamic form-object processing. Those who do classical psychophysics studies might aim to replicate our results and members of the neuroimaging community might employ a similar masking paradigm to assess fMRI activation.

## Author Contributions

EG designed the study, provided custom test equipment, experimental apps, and oversaw drafting of the manuscript. AG tested respondents, evaluated the data, did the literature review, and provided various drafts of the manuscript.

## Conflict of Interest Statement

The authors declare that the research was conducted in the absence of any commercial or financial relationships that could be construed as a potential conflict of interest.
